# Supplementary figures and images for: Effectiveness of antiresorptive medications in women on long-term dialysis after hip fracture: A population-based cohort study
Source: PLoS One. 2020 Sep 2;15(9):e0238248. doi: 10.1371/journal.pone.0238248 (PMC7467303; doi:10.1371/journal.pone.0238248)

S1 Fig. Persistence rates of alendronate and raloxifene


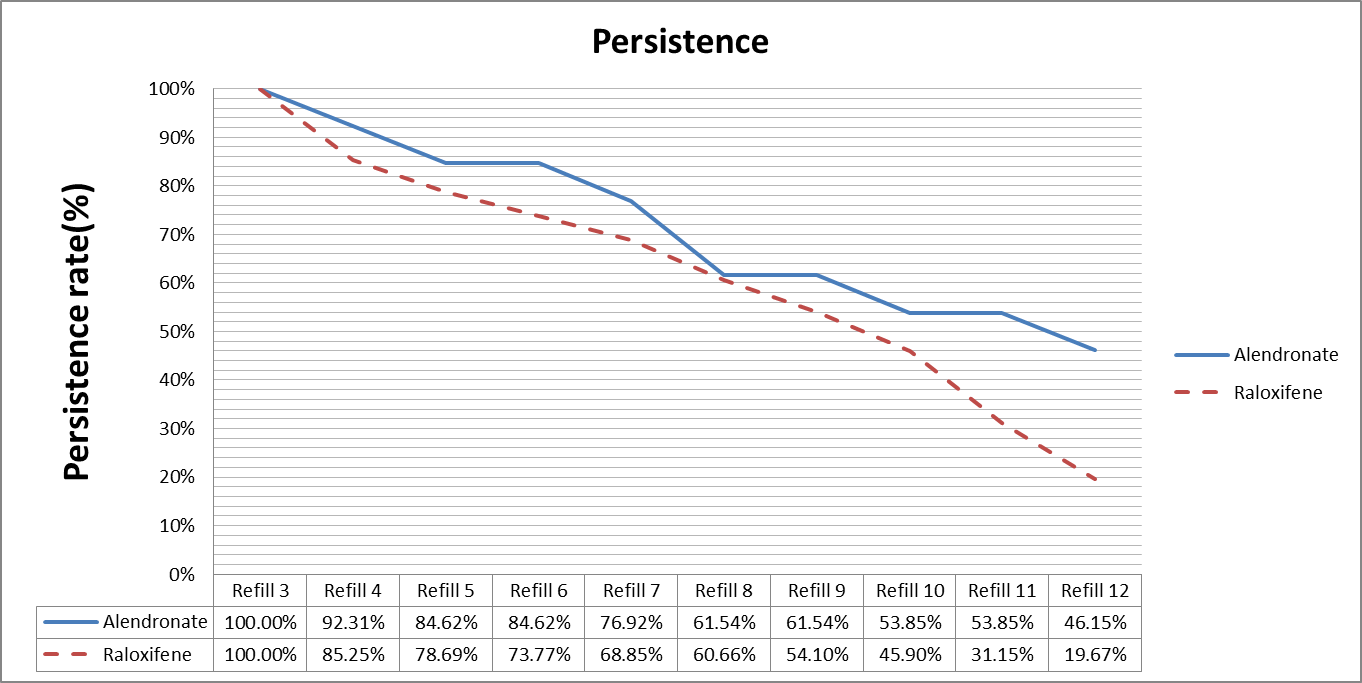

Supplement: S1 Fig — (DOCX) [file pone.0238248.s001.docx]
