# Supplementary material for: Effectiveness of antiresorptive medications in women on long-term dialysis after hip fracture: A population-based cohort study
Source: PLoS One. 2020 Sep 2;15(9):e0238248. doi: 10.1371/journal.pone.0238248 (PMC7467303; doi:10.1371/journal.pone.0238248)
Supplement: S1 Table — (DOCX) [file pone.0238248.s002.docx]

S1Table. Corresponding ICD-9-CM codes used for the chronic conditions /comorbidities in this study

| Chronic conditions/Comorbidities | ICD-9-CM diagnosis codes/Medical code |
| --- | --- |
| Osteoporosis | 733.0 |
| Fracture history^a^ | 812.0, 812.2, 812.4, 733.11, 808.0, 808.2, 808.4, 808.8, 810.0, 807.0, 821.0, 821.2, 823.0, 823.2, 823.8, 733.15, 733.16. |
| Renal osteodystrophy | 588.0 |
| Hyperparathyroidism | 252.0 |
| Peripheral neuropathy | 356,357.1,357.3 – 357.9 |
| Parkinson’s disease | 332 |
| Cardiovascular disease | 410–414; MI: 410.XX; CHF:428; Arrhythmia:426–427 |
| Cerebrovascular disease | 430-437 |
| Diabetes mellitus | 250 |
| Hypertension | 401-405 |
| Chornic pulmonary disease | Asthma: 493; COPD:490-492,496 |
| Cataracts | 366 |
| Dementia | 290; Alzheimer’s disease: 331.0 |
| Mental disorders | Depression: 296.2, 296.3, 300.4, 311 |
| Rheumatic arthritis | 714 |
| Chronic liver disease | 571 |

Abbreviation: MI, myocardial infarction; CHF, congestive heart failure; COPD, chronic obstruction pulmonary disease

^a^Non-vertebral fractures other than radius/ulna or hip fracture (include: pelvis, clavicle, rib, femur, leg, humerus)
