# Supplementary material for: Effectiveness of antiresorptive medications in women on long-term dialysis after hip fracture: A population-based cohort study
Source: PLoS One. 2020 Sep 2;15(9):e0238248. doi: 10.1371/journal.pone.0238248 (PMC7467303; doi:10.1371/journal.pone.0238248)
Supplement: S2 Table — (DOCX) [file pone.0238248.s003.docx]

S2 Table. Corresponding ATC codes used for the comedications in this study

| Comedication | ATC code |
| --- | --- |
| Glucocorticoids | H02AB01, H02AB02, H02AB04, H02AB05, H02AB06, H02AB08, H02AB09, H02AB10 |
| Antiepileptic drugs | N03AA02, N03AA03, N03AB02, N03AF01, N03AF02, N03AG01, N03AG04, N03AG06, N03AX09, N03AX11, N03AX12, N03AX14, N03AX15, N03AX16, N03AX18, N03AX22 |
| Anti-depressants^a^ | N06AA02, N06AA04, N06AA09, N06AA12, N06AA14, N06AA21, N06AB03, N06AB04, N06AB05, N06AB06, N06AB08, N06AB10, N06AG02, N06AX01, N06AX02, N06AX03, N06AX05, N06AX09, N06AX11, N06AX12, N06AX16, N06AX17, N06AX21, N06AX22 |
| Beta-blocking agents | C07AA91, C07AA02, C07AA03, C07AA05, C07AA06, C07AA07, C07AA12, C07AA15, C07AA19, C07AB02, C07AB03, C07AB04, C07AB05, C07AB07, C07AB09, C07AG01, C07AG02, C07BA68, C07BB02, C07BB03, C07AB07, C07AB09, C07AG01, C07AG02, C07BA68, C07BB02, C07BB03 |
| Benzodiazepines | N05CD01, N05CD02, N05CD03, N05CD04, N05CD05, N05CD06, N05CD07, N05CD08, N05CD09, N05AE01, N05BA01, N05BA02, N05BA03, N05BA04, N05BA06, N05BA08, N05BA09, N05BA11, N05BA12, N05BA16, N05BA17, N05BA22 |
| Analgesics | N02AA01, N02AA02, N02AA03, N02AA05, N02AB03, N02AE01, N02AF02, N02AX02, N02BA01, N02BA04, N02BA05, N02BA06, N02BA07, N02BA11, N02BB01, N02BB04, N02BE04, N02BG03, N02BG06 |
| Sedatives and Hypnotics | N05CA01, N05CA02, N05CA06, N05CA21, N05CA22, N05CE01, N05CF01, N05CF02, N05CF03, N05CF04, N05CM03, N05CM05, N05CM12, N05CM18 |
| Statins | C10AA01, C10AA02, C10AA03, C10AA04, C10AA05, C10AA07, C10AA08 |
| Calcitriol therapy | A11CC, A11CC03, A11CC04 |
| Diuretics^b^ | C03AA01, C03AA03, C03AA06, C03AA07, C03AA91, C03CA01, C03CA02, C03CC01 |
| Oral diabetes medications | A10BA02, A10BA03, A10BB01, A10BB02, A10BB03, A10BB04, A10BB05, A10BB07, A10BB08, A10BB09, A10BB12, A10BB31, A10BD, A10BD02, A10BD03, A10BD05, A10BD07, A10BD08, A10BD10, A10BD11, A10BD13, A10BF01, A10BF02, A10BG02, A10BG03, A10BH01, A10BH02, A10BH03, A10BH04, A10BH05, A10BJ01, A10BJ02, A10BX01, A10BX02, A10BX03, A10BX08 |
| Proton pump inhibitors | A02BC01, A02BC02, A02BC03, A02BC04, A02BC05, A02BC06 |
| Nitrates | C01DA, C01DA02, C01DA08, C01DA52 |
| Oral NSAIDs | M01A, M01AA01, M01AB01, M01AB02, M01AB03, M01AB05, M01AB06, M01AB08, M01AB11, M01AB15, M01AB16, M01AC, M01AC01, M01AC02, M01AC06, M01AE01, M01AE02, M01AE03, M01AE04, M01AE05, M01AE09, M01AE11, M01AE16, M01AE52, M01AG, M01AG01, M01AG02, M01AG03, M01AG04, M01AH01, M01AH02, M01AH05, M01AX01, M01AX02, M01AX07, M01AX17, M01AX91 |

Abbreviation:ATC, Anatomical therapeutic chemical, SSRI, Selective serotonin reuptake inhibitors; NSAIDs, nonsteroidal anti-inflammatory drugs;

^a^any type antidepressants, SSRI antidepressants, tricyclic antidepressants, miscellaneous ADs.

^b^thiazide, loop diuretics.
