# Supplementary material for: Effectiveness of antiresorptive medications in women on long-term dialysis after hip fracture: A population-based cohort study
Source: PLoS One. 2020 Sep 2;15(9):e0238248. doi: 10.1371/journal.pone.0238248 (PMC7467303; doi:10.1371/journal.pone.0238248)
Supplement: S3 Table — (DOCX) [file pone.0238248.s004.docx]

S3 Table. Significant covariates of baseline characteristics in univariate Cox-regression

| **Risk of hospitalization for second hip fracture in AR users versus non-users and raloxifene versus alendronate** | | | |
| --- | --- | --- | --- |
| Treatment groups | Covariates with criterion P value<0.1 | | |
|  | Before propensity score matching (M1 model) | | Propensity score matching (M2 model) |
| AR non-users versus AR users | Age, Fracture history, Socioeconomic, Peripheral neuropathy, Analgesics, Oral NSAIDs, Beta-blocking agents | | Age, Fracture history, Diabetes mellitus, Beta-blocking agents |
| Raloxifene versus Alendronate | Age, Fracture history, Calcitriol, Beta-blocking agents | | Age, Fracture history, Calcitriol, Beta-blocking agents |
| **1-year and 2-year mortality of AR non-users versus users and raloxifene versus alendronate** | | | |
| Treatment groups | Covariates with criterion P value <0.1 | | |
|  | Before propensity score matching (M1 model) | Propensity score matching (M2 model) | |
| AR non-users versus AR users | | | |
| 1-year mortality | Age, Fracture history, Cardiovascular disease, Cataracts, Glucocorticoids, Anti-depressants | Age, Peripheral neuropathy, Glucocorticoids | |
| 2-year mortality | Age, Fracture history, Duration of dialysis, Socioeconomic, Cardiovascular disease, Cataracts, Sedatives and Hypnotics, Diuretics | Age | |
| Raloxifene versus Alendronate | | | |
| 1-year mortality | Parkinson’s disease | Parkinson’s disease | |
| 2-year mortality | Diabetes mellitus | Diabetes mellitus | |
