# Supplementary material for: Effectiveness of antiresorptive medications in women on long-term dialysis after hip fracture: A population-based cohort study
Source: PLoS One. 2020 Sep 2;15(9):e0238248. doi: 10.1371/journal.pone.0238248 (PMC7467303; doi:10.1371/journal.pone.0238248)
Supplement: S4 Table — (DOCX) [file pone.0238248.s005.docx]

S4 Table. The PDC stratification and outcomes between AR users/non-users and treatment group

|  | Hazard Ratio(95%CI) | | | | | | |
| --- | --- | --- | --- | --- | --- | --- | --- |
|  | Risk of hospitalization for secondary hip fracture | | | 1-year mortality^#^ | | 2-year mortality^#^ | |
|  | Adjusted M1^a^ | P value | | Adjusted M1 | P value | Adjusted M1 | P value |
| AR users versus AR non-users | | | | | | | |
| AR non-users | 1.00 (Reference) |  | - | | - | 1.00 (Reference) |  |
| AR users, PDC<0.5 | 0.33 (0.04-2.88) | 0.32 | - | | - | 0.68 (0.30-1.61) | 0.33 |
| AR users, 0.5≤ PDC<0.8 | 1.62 (0.35-7.42) | 0.54 | - | | - | 0.09 (0.02-0.63) | <0.05 |
| AR users, PDC≥0.8 | 0.50 (0.09-2.78) | 0.42 | - | | - | 0.26 (0.06-1.12) | 0.07 |
| Treatment group |  |  |  | |  |  |  |
| PDC<0.5 | 1.00 (Reference) |  | - | | - | 1.00 (Reference) |  |
| 0.5≤ PDC<0.8 | 4.16 (0.37-46.90) | 0.25 | - | | - | 0.12 (0.02-0.95) | <0.05 |
| PDC≥0.8 | 3.33 (0.19-58.14) | 0.41 | - | | - | 0.36 (0.08-1.69) | 0.19 |
| Alendronate groups |  |  |  | |  |  |  |
| PDC<0.5 | **-** | **-** | - | | - | - | - |
| 0.5≤ PDC<0.8 | **-** | **-** | - | | - | - | - |
| PDC≥0.8 | **-** | **-** | - | | - | - | - |
| Raloxifene groups |  |  |  | |  |  |  |
| PDC<0.5 | 1.00 (Reference) |  | - | | - | 1.00 (Reference) |  |
| 0.5≤ PDC<0.8 | 2.39 (0.22-25.75) | 0.47 | - | | - | 0.16 (0.02-1.28) | 0.08 |
| PDC≥0.8 | 2.77 (0.25-31.17) | 0.41 | - | | - | 0.45 (0.09-2.18) | 0.32 |

Abbreviation: AR, Antiresorptive medications; PDC, proportion of days covered.

Notes: M1: After propensity score matching, adjusted with significant covariates of baseline characteristics in univariate Cox-regression (p<0.1) (S3 Table).

^a^: covariates in M1, age, fracture history. ^#^: time-varying adjusted failure.
