# Supplementary material for: Effectiveness of antiresorptive medications in women on long-term dialysis after hip fracture: A population-based cohort study
Source: PLoS One. 2020 Sep 2;15(9):e0238248. doi: 10.1371/journal.pone.0238248 (PMC7467303; doi:10.1371/journal.pone.0238248)
Supplement: S5 Table — (DOCX) [file pone.0238248.s006.docx]

S5 Table. The refilled times stratification and outcomes between AR users/non-users and treatment group

|  | Hazard Ratio (95%CI) | | | | | | |
| --- | --- | --- | --- | --- | --- | --- | --- |
|  | Risk of hospitalization for secondary hip fracture | | 1-year mortality^#^ | | 2-year mortality^#^ | |  |
|  | Adjusted M1 | P value | Adjusted M1 | P value | Adjusted M1 | P value |  |
| AR users versus AR non-users | | | | | | |  |
| AR non-users | 1.00 (Reference) |  | - | - | 1.00 (Reference) |  |  |
| Refill ≥ 3 times | 0.32 (0.03-2.89) | 0.31 | - | - | 0.56 (0.25-1.25) | 0.16 |  |
| Refill ≥ 6 times | 1.25 (0.22-7.24) | 0.81 | - | - | 0.15 (0.02-1.14) | 0.07 |  |
| Refill ≥ 9 times | 0.69 (0.14-3.32) | 0.64 | - | - | 0.19 (0.05-0.82) | <0.05 |  |
| Alendronate groups |  |  |  |  |  |  |  |
| Refill ≥ 3 times | - | - | - | - | - | - |  |
| Refill ≥ 6 times | - | - | - | - | - | - |  |
| Refill ≥ 9 times | - | - | - | - | - | - |  |
| Raloxifene groups |  |  | - | - |  |  |  |
| Refill ≥ 3 times | 1.00 (Reference) |  | - | - | 1.00 (Reference) |  |  |
| Refill ≥ 6 times | 3.67 (0.32-42.17) | 0.30 | - | - | 0.22 (0.03-1.80) | 0.16 |  |
| Refill ≥ 9 times | 3.00 (0.26-34.49) | 0.38 | - | - | 0.31 (0.06-1.52) | 0.15 |  |

Abbreviation: AR, Antiresorptive medications.

Notes: M1: After propensity score matching, adjusted with significant covariates of baseline characteristics in univariate Cox-regression (p<0.1) (S3 Table). ^#^: time-varying adjusted failure.
