# Supplementary material for: Effectiveness of antiresorptive medications in women on long-term dialysis after hip fracture: A population-based cohort study
Source: PLoS One. 2020 Sep 2;15(9):e0238248. doi: 10.1371/journal.pone.0238248 (PMC7467303; doi:10.1371/journal.pone.0238248)
Supplement: S6 Table — (DOCX) [file pone.0238248.s007.docx]

S6 Table. Subgroup analysis of outcomes between AR users/non-users and alendronate/raloxifene

| Hazard Ratio (95% CI) | | | | | | | |
| --- | --- | --- | --- | --- | --- | --- | --- |
|  | Risk of hospitalization for secondary hip fracture | | 1-year mortality^#^ | | | 2-year mortality^#^ | |
|  | Adjusted M1 | P value | Adjusted M1 | | P value | Adjusted M1 | P value |
| *Stratified by age groups (years)* | | | | | | | |
| AR users versus AR non-users (reference group) | | | | | | | |
| 50-64 | 0.46 (0.05-3.88)^#^ | 0.47 | | - | - | - | - |
| 65-79 | 0.35 (0.01-14.29)^#^ | 0.58 | | 0.47 (0.09-2.55) | 0.38 | 0.36 (0.15-0.87) | <0.05 |
| ≥ 80 | 0.17 (0.01-3.90)^#^ | 0.26 | | 0.50 (0.08-2.97) | 0.44 | 0.50 (0.13-1.87) | 0.30 |
| Raloxifene versus Alendronate (reference group) | | | | | | | |
| 50-64 | 1.12 (0.10-13.12)^#^ | 0.92 | - | | - | - | - |
| 65-79 | - | - | 0.33 (0.02-6.39) | | 0.46 | 1.08 (0.13-8.99) | 0.94 |
| ≥ 80 | - | - | - | | - | - | - |
| *Stratified by socioeconomic status, New Taiwan Dollar* | | | | | | | |
| AR users versus AR non-users (reference group) | | | | | | | |
| < 15,840 | 0.49(0.16-3.84) | 0.77 | 0.34 (0.09-1.32) | | 0.12 | 0.34 (0.14-0.87) | <0.05 |
| ≥ 15,840 | 0.19(0.01-4.14) | 0.30 | 0.31 (0.03-3.38) | | 0.33 | - | - |
| Raloxifene versus Alendronate (reference group) | | | | | | | |
| < 15,840 | - | - | 0.25 (0.01-4.40) | | 0.34 | 1.19 (0.13-10.71) | 0.87 |
| ≥ 15,840 | 3.38 (0.06-196.00) | 0.56 | - | | - | - | - |

Abbreviation: AR, Antiresorptive medications.

Notes: M1: After propensity score matching, adjusted with significant covariates of baseline characteristics in univariate Cox-regression (p<0.1) (S3 Table). ^#^: time-varying adjusted failure.

S6 Table. Subgroup analysis of outcomes between AR users/non-users and alendronate/raloxifene (continued)

| Hazard Ratio (95% CI) | | | | | | | |
| --- | --- | --- | --- | --- | --- | --- | --- |
|  | Risk of hospitalization for secondary hip fracture | | 1-year mortality^#^ | | | 2-year mortality^#^ | |
|  | Adjusted M1 | P value | Adjusted M1 | | P value | Adjusted M1 | P value |
| *Stratified by duration of dialysis,* *months* | | | | | | | |
| AR users versus AR non-users (reference group) | | | | | | | |
| < 24 | 2.30 (0.21-25.13) | 0.49 | | 1.27 (0.16-9.90) | 0.81 | 0.21 (0.06-0.77) | <0.05 |
| ≥ 24 | 0.14 (0.01-2.22) | 0.16 | | 0.42 (0.07-2.44) | 0.34 | 0.52 (0.22-1.25) | 0.14 |
| Raloxifene versus Alendronate (reference group) | | | | | | | |
| < 24 | - | - | - | | - | - | - |
| ≥ 24 | - | - | - | | - | - | - |
| *Stratified by previous facture history* | | | | | | | |
| AR users versus AR non-users (reference group) | | | | | | | |
| Any fracture history | 0.60 (0.03-11.75)^#^ | 0.74 | 0.12 (0.01-2.83) | | 0.19 | 0.15 (0.03-0.75) | <0.05 |
| No fracture history | 0.86 (0.22-3.32) | 0.82 | 0.46 (0.11-1.91) | | 0.28 | 0.37 (0.16-0.87) | <0.05 |
| Raloxifene versus Alendronate (reference group) | | | | | | | |
| Any fracture history | - | - | - | | - | - | - |
| No fracture history | 1.62 (0.14-18.94) | 0.70 | 0.26 (0.02-3.35) | | 0.30 | 1.31 (0.16-10.86) | 0.80 |

Abbreviation: AR, Antiresorptive medications.

Notes: M1: After propensity score matching, adjusted with significant covariates of baseline characteristics in univariate Cox-regression (p<0.1) (S3 Table). ^#^: time-varying adjusted failure.

S6 Table. Subgroup analysis of outcomes between AR users/non-users and alendronate/raloxifene(continued)

| Hazard Ratio (95% CI) | | | | | | | | | | | |
| --- | --- | --- | --- | --- | --- | --- | --- | --- | --- | --- | --- |
|  | Risk of hospitalization for secondary hip fracture | | 1-year mortality^#^ | | | | | 2-year mortality^#^ | | | |
|  | Adjusted M1^a^ | P value | Adjusted M1 | | | P value | | Adjusted M1 | | P value | |
| *Stratified by previous steroid use* | | | | | | | | | | | |
| AR users versus AR non-users (reference group) | | | | | | | | | | | |
| No steroid | 0.69 (0.16-3.02) | 0.62 | | 0.87 (0.21-3.56) | 0.85 | | 0.49 (0.23-1.03) | | 0.06 | |  |
| Steroid | - | - | | - | - | | - | | - | |  |
| Raloxifene versus Alendronate (reference group) | | | | | | | | | | | |
| No steroid | - | - | - | | | - | | - | | - | |
| Steroid | 1.45 (0.08-26.79) | 0.80 | 0.85 (0.06-11.69) | | | 0.91 | | 1.06 (0.13-8.55) | | 0.96 | |
| *Stratified by cardiovascular disease* | | | | | | | | | | | |
| AR users versus AR non-users (reference group) | | | | | | | | | | | |
| Yes | 1.48 (0.14-16.11) | 0.75 | 0.33 (0.06-1.69) | | | 0.18 | | 0.40 (0.14-1.14) | | 0.09 | |
| No | 0.33 (0.03-3.83) | 0.38 | 0.47 (0.06-3.59) | | | 0.46 | | 0.30 (0.11-0.83) | | <0.05 | |
| Raloxifene versus Alendronate (reference group) | | | | | | | | | | | |
| Yes | 0.86 (0.07-11.27)^#^ | 0.91 | 0.05 (0.00-1.47) | | | 0.08 | | 0.34 (0.03-4.38) | | 0.41 | |
| No | - | - | - | | | - | | - | | - | |

Abbreviation: AR, Antiresorptive medications.

Notes: M1: After propensity score matching, adjusted with significant covariates of baseline characteristics in univariate cox-regression (p<0.1) (S3 Table). ^#^: time-varying adjusted failure.
