# Supplementary material for: Effectiveness of antiresorptive medications in women on long-term dialysis after hip fracture: A population-based cohort study
Source: PLoS One. 2020 Sep 2;15(9):e0238248. doi: 10.1371/journal.pone.0238248 (PMC7467303; doi:10.1371/journal.pone.0238248)
Supplement: S7 Table — (DOCX) [file pone.0238248.s008.docx]

S7 Table. Sensitivity analysis of hospitalization for secondary hip fracture: excluded short-term users

| Outcome | Events,  N (%) | Fracture rate, per 100 PYs | Hazard Ratio (95% CI) | | | | | | | | |
| --- | --- | --- | --- | --- | --- | --- | --- | --- | --- | --- | --- |
|  |  |  | Crude | P value | Adjusted M1 | P value | | Adjusted M2 | P value | Adjusted M3 | P value |
| AR users versus AR non-users | | | | | | | | | | | |
| AR non-users | 44 (4.98) | 2.08 | 1.00 (Reference) |  | 1.00 (Reference) |  | | 1.00 (Reference) |  | 1.00 (Reference) |  |
| AR users | 6 (8.33) | 2.89 | 1.38 (0.59-3.26) | 0.45 | 1.20 (0.48-3.04) | 0.69 | | 0.44 (0.10-2.03) | 0.30 | 0.55 (0.12-2.54) | 0.44 |
| Raloxifene versus Alendronate | | | | | | | | | | | |
| Alendronate | 1 (8.33) | 2.92 | 1.00 (Reference) |  | 1.00 (reference) | |  | 1.00 (reference) |  | 1.00 (Reference) |  |
| Raloxifene | 5 (8.47) | 3.02 | 0.97 (0.11-8.41) | 0.98 | 1.59 (0.10-26.32) | | 0.75 | 1.59 (0.10-26.32) | 0.75 | 1.63 (0.58-4.61) | 0.36 |
| Alendronate versus AR non-users | | | | | | | | | | | |
| AR non-users | 44 (4.98) | 2.08 | 1.00 (Reference) |  | 1.00 (Reference) | |  | 1.00 (Reference) |  | 1.00 (Reference) |  |
| Alendronate | 1 (8.33) | 2.92 | 1.36 (0.19-9.90) | 0.76 | 1.16 (0.07-19.68) | | 0.92 | 0.14 (0.00-5.59) | 0.29 | 0.16 (0.02-1.49) | 0.11 |
| Raloxifene versus AR non-users | | | | | | | | | | | |
| AR non-users | 44 (4.98) | 2.08 | 1.00 (Reference) |  | 1.00 (Reference) | |  | 1.00 (Reference) |  | 1.00 (Reference) |  |
| Raloxifene | 5 (10.20) | 3.02 | 1.47 (0.58-3.70) | 0.42 | 1.27 (0.47-3.41) | | 0.64 | 0.48 (0.11-2.06) | 0.32 | 0.53 (0.13-2.20) | 0.38 |

Abbreviation: AR, Antiresorptive medications; Pys, person-years.

Notes: M1: Before propensity score matching, adjusted with significant covariates of baseline characteristics in univariate Cox-regression (p<0.1) (S3 Table) and age, fracture history; M2: Propensity score matching, adjusted with significant covariates of baseline characteristics in univariate Cox-regression (p<0.1) (S3 Table); M3: Adjusted for all variables in M2 and competing risk. ^#^: time-varying adjusted failure.
