# Supplementary material for: Effectiveness of antiresorptive medications in women on long-term dialysis after hip fracture: A population-based cohort study
Source: PLoS One. 2020 Sep 2;15(9):e0238248. doi: 10.1371/journal.pone.0238248 (PMC7467303; doi:10.1371/journal.pone.0238248)
Supplement: S8 Table — (DOCX) [file pone.0238248.s009.docx]

S8 Table. Sensitivity analysis: intention-to-treat scenario

| Hazard Ratio (95% CI) | | | | | | |
| --- | --- | --- | --- | --- | --- | --- |
|  | Risk of hospitalization for secondary hip fracture | | 1-year mortality^#^ | | 2-year mortality^#^ | |
|  | Adjusted M1^a^ | P value | Adjusted M1 | P value | Adjusted M1 | P value |
| *Analyzed by Intention-to-treat definition* | | | | | | |
| AR users versus AR non-users | | | | | | |
| AR non-users | 1.00 (Reference) |  | 1.00 (Reference) |  | 1.00 (Reference) |  |
| AR users | 0.60 (0.16-2.20) | 0.44 | 0.26 (0.10-0.70) | <0.05 | 0.36 (0.20-0.65) | <0.05 |
| Raloxifene versus Alendronate | | | | | | |
| Alendronate | 1.00 (Reference) |  | 1.00 (Reference) |  | 1.00 (Reference) |  |
| Raloxifene | 2.66 (0.28-25.70)^#^ | 0.40 | 0.84 (0.08-8.55) | 0.89 | 2.09 (0.27-16.35) | 0.48 |
| Alendronate versus AR non-users | | | | | | |
| AR non-users | 1.00 (Reference) |  | 1.00 (Reference) |  | 1.00 (Reference) |  |
| Alendronate | 0.27 (0.01-11.14) | 0.49 | 0.17 (0.02-1.51) | 0.11 | 0.15 (0.02-1.10) | 0.06 |
| Raloxifene versus AR non-users | | | | | | |
| AR non-users | 1.00 (Reference) |  | 1.00 (Reference) |  | 1.00 (Reference) |  |
| Raloxifene | 0.59 (0.15-2.36) | 0.46 | 0.26 (0.07-0.995) | <0.05 | 0.37 (0.17-0.77) | <0.05 |

Abbreviation: AR, Antiresorptive medications.

Notes: M1: After propensity score matching, adjusted with significant covariates of baseline characteristics in univariate cox-regression (p<0.1) (S3 Table). ^a^: covariates in M1, age, fracture history. ^#^: time-varying adjusted failure.
