# Supplementary material for: Effectiveness of antiresorptive medications in women on long-term dialysis after hip fracture: A population-based cohort study
Source: PLoS One. 2020 Sep 2;15(9):e0238248. doi: 10.1371/journal.pone.0238248 (PMC7467303; doi:10.1371/journal.pone.0238248)
Supplement: S9 Table — (DOCX) [file pone.0238248.s010.docx]

S9 Table. Sensitivity analysis: non-change group

| Hazard Ratio (95% CI) | | | | | | | |
| --- | --- | --- | --- | --- | --- | --- | --- |
|  | Risk of hospitalization for secondary hip fracture | | 1-year mortality^#^ | | | 2-year mortality^#^ | |
|  | Adjusted M1 | P value | Adjusted M1 | | P value | Adjusted M1 | P value |
| *Analyzed by non-change group* | | | | | | | |
| AR users versus AR non-users | | | | | | | |
| AR non-users | 1.00 (Reference) |  | | 1.00 (Reference) |  | 1.00 (Reference) |  |
| AR users | 0.60 (0.16-2.20) | 0.44 | | 0.26 (0.10-0.70) | <0.05 | 0.36 (0.20-0.65) | <0.05 |
| Raloxifene versus Alendronate | | | | | | | |
| Alendronate | 1.00 (Reference) |  | 1.00 (Reference) | |  | 1.00 (Reference) |  |
| Raloxifene | 3.43 (0.15-81.33)^#^ | 0.45 | 0.83 (0.08-8.47) | | 0.88 | 1.93 (0.25-15.22) | 0.53 |
| Alendronate versus AR non-users | | | | | | | |
| AR non-users | 1.00 (Reference) |  | 1.00 (Reference) | |  | 1.00 (Reference) |  |
| Alendronate | 0.28 (0.01-11.36) | 0.50 | 0.17 (0.02-1.55) | | 0.12 | 0.18 (0.02-1.29) | 0.09 |
| Raloxifene versus AR non-users | | | | | | | |
| AR non-users | 1.00 (Reference) |  | 1.00 (Reference) | |  | 1.00 (Reference) |  |
| Raloxifene | 0.53 (0.12-2.29) | 0.39 | 0.29 (0.08-1.07) | | 0.06 | 0.42 (0.20-0.88) | <0.05 |

Abbreviation: AR: Antiresorptive medications.

Notes: M1: After propensity score matching, adjusted with significant covariates of baseline characteristics in univariate Cox-regression (p<0.1) (S3 Table). ^#^ : time-varying adjusted failure.
