# Supplementary material for: Effectiveness of antiresorptive medications in women on long-term dialysis after hip fracture: A population-based cohort study
Source: PLoS One. 2020 Sep 2;15(9):e0238248. doi: 10.1371/journal.pone.0238248 (PMC7467303; doi:10.1371/journal.pone.0238248)
Supplement: S10 Table — (DOCX) [file pone.0238248.s011.docx]

S10 Table. Sensitivity analysis: primary analysis plus 90 days

| Hazard Ratio (95% CI) | | | | | | | |
| --- | --- | --- | --- | --- | --- | --- | --- |
|  | Risk of hospitalization for secondary hip fracture | | 1-year mortality^#^ | | | 2-year mortality^#^ | |
|  | Adjusted M1 | P value | Adjusted M1 | | P value | Adjusted M1 | P value |
| *Analyzed by primary analysis plus 90 days* | | | | | | | |
| AR users versus AR non-users | | | | | | | |
| AR non-users | 1.00(reference) |  | | 1.00 (reference) |  | 1.00 (reference) |  |
| AR users | 0.76 (0.22-2.62) | 0.66 | | 0.74 (0.26-2.14) | 0.58 | 0.48 (0.25-0.91) | <0.05 |
| Raloxifene versus Alendronate | | | | | | | |
| Alendronate | 1.00 (reference) |  | 1.00 (reference) | |  | 1.00 (reference) |  |
| Raloxifene | 1.66 (0.16-17.49) | 0.68 | 0.62 (0.06-6.63) | | 0.69 | 0.37 (0.02-5.94) | 0.62 |
| Alendronate versus AR non-users | | | | | | | |
| AR non-users | 1.00 (Reference) |  | 1.00 (Reference) | |  | 1.00 (Reference) |  |
| Alendronate | 1.30 (0.14-11.91) | 0.81 | 0.39 (0.04-3.48) | | 0.40 | 0.35 (0.08-1.50) | 0.16 |
| Raloxifene versus AR non-users | | | | | | | |
| AR non-users | 1.00 (Reference) |  | 1.00 (Reference) | |  | 1.00 (Reference) |  |
| Raloxifene | 0.70 (0.18-2.70) | 0.60 | 0.77 (0.26-2.29) | | 0.63 | 0.51 (0.25-1.01) | 0.05 |

Abbreviation: AR, Antiresorptive medications.

Notes: M1: After propensity score matching, adjusted with significant covariates of baseline characteristics in univariate Cox-regression (p<0.1) (S3 Table). ^#^: time-varying adjusted failure.
