# Supplementary material for: Effectiveness of antiresorptive medications in women on long-term dialysis after hip fracture: A population-based cohort study
Source: PLoS One. 2020 Sep 2;15(9):e0238248. doi: 10.1371/journal.pone.0238248 (PMC7467303; doi:10.1371/journal.pone.0238248)
Supplement: S11 Table — (DOCX) [file pone.0238248.s012.docx]

S11 Table. Sensitivity analysis: immortal time and lag time adjustment

| Hazard Ratio (95% CI) | | | | | | | |
| --- | --- | --- | --- | --- | --- | --- | --- |
|  | Risk of hospitalization for secondary hip fracture | | 1-year mortality^#^ | | | 2-year mortality^#^ | |
|  | Adjusted M1 | P value | Adjusted M1 | | P value | Adjusted M1 | P value |
| *Analyzed by primary analysis plus 90 days* | | | | | | | |
| AR users versus AR non-users | | | | | | | |
| AR non-users | 1.00 (Reference) |  | | 1.00 (Reference) |  | 0.28(0.02-4.60) | 0.37 |
| AR users | 0.27 (0.06-1.21) | 0.09 | | 0.18 (0.05-0.77) | <0.05 | 0.17 (0.06-0.50) | <0.05 |
| Raloxifene versus Alendronate | | | | | | | |
| Alendronate | - | - | - | | - | 1.00 (Reference) |  |
| Raloxifene | - | - | - | | - | 0.44 (0.04-4.93) | 0.50 |

Abbreviation: AR, Antiresorptive medications. Notes: M1: After propensity score matching, adjusted with significant covariates of baseline characteristics in univariate Cox-regression (p<0.1) (S3 Table), immortal time, and lag time.
