# Supplementary material for: Effectiveness of antiresorptive medications in women on long-term dialysis after hip fracture: A population-based cohort study
Source: PLoS One. 2020 Sep 2;15(9):e0238248. doi: 10.1371/journal.pone.0238248 (PMC7467303; doi:10.1371/journal.pone.0238248)
Supplement: Attachment — Submitted filename: Response to Reviewers_1090811.docx [file pone.0238248.s014.docx]

Reviewer #1: Thank you for addressing comments from reviewers so thoroughly. I have only a few comments / clarifications.

1. I suggest you reword the following updated sentence (in the Statistical analysis, page 7, paragraph 1). "To possibly obtain sufficient statistical power...." replace that with "To increase statistical power". This might just be poor English, but it's not correct.

Ans: Thank you for pointing out the correct description. We have corrected this error in the new version.

On line 12-14, page 7:
“To increase statistical power, PS matching pair from 1:1 to 1:5 using the Greedy 5 to 1 digit technique [15] was taken into consideration.”

2. In the discussion section, (page 19, paragraph 2), a sentence reads "First, the small sample size in our study may led to a lack of statistical significance". This, too, may be poor English expression (I note that the original manuscript has been edited by an native English speaker) but this is not correct either. I presume the authors mean that the number of events observed was low, leading to inaccurate estimates of effect size.

Ans: Thank you for the valuable suggestion. We have made additional English editing by another native English speaker. This error has been corrected in the new version.

On line 30-32, page 30:

“Firstly, the sample size was small, though it was representative, and the information in Taiwan’s dialysis database is relatively more complete than that found in other countries.”
